# Senescent Microglia: The Key to the Ageing Brain?

**DOI:** 10.3390/ijms22094402

**Published:** 2021-04-22

**Authors:** Eleanor K. Greenwood, David R. Brown

**Affiliations:** Department of Biology and Biochemistry, University of Bath, Bath BA2 7AY, UK; ekg39@bath.ac.uk

**Keywords:** microglia, senescence, iron, ageing, neurodegeneration, Senescence Associated Secretory Phenotype

## Abstract

Ageing represents the single biggest risk factor for development of neurodegenerative disease. Despite being such long-lived cells, microglia have been relatively understudied for their role in the ageing process. Reliably identifying aged microglia has proven challenging, not least due to the diversity of cell populations, and the limitations of available models, further complicated by differences between human and rodent cells. Consequently, the literature contains multiple descriptions and categorisations of microglia with neurotoxic phenotypes, including senescence, without any unifying markers. The role of microglia in brain homeostasis, particularly iron storage and metabolism, may provide a key to reliable identification.

## 1. Introduction

Microglia, far from being simply ‘brain glue’, play an important role as the brain’s resident immune cells [1,2]. Their roles include phagocytic clearance of debris [3], pruning of synapses [4], and possibly even contributing to synaptic activity [5], being of critical importance from early development to ageing. Microglia are an extremely heterogeneous population, within the brain [6,7,8], between sexes [9], but also between species, with microglia derived from mice showing significant differences to human-derived microglia in transcriptomic studies [10,11]. The protein Ionised Calcium Binding Adaptor Molecule 1 (Iba1) is recognised as a specific microglia marker [12], allowing them to be distinguished from other glial cells, although not from macrophages and monocytes [13].

This review will examine the diverse range of microglial phenotypes, in particular, senescent microglia and their role in neurodegenerative disease. The importance of reliable markers in furthering attempts to target and eliminate harmful microglia will also be discussed, along with the key role played by accurate models of microglial senescence. Various models of microglial senescence will be evaluated, in particular, iron supplementation and its relation to physiological senescence. This review will highlight the importance of developing accurate, reliable markers, but also some of the challenges such a task presents.

## 2. Microglial Origins

Microglia are distinct from other cell types in the brain in that they are derived not from the neural tube, but from primitive macrophages in the yolk sac, and not simply from the bone marrow, as originally suggested [1,2,3]. They are maintained and renewed from the population within the brain [4]. However, unlike circulating monocytes, the master transcription factor PU.1 has been found to be critical for microglial development, as they are entirely absent in PU.1 knockout mice [5,6]. Other key factors for microglial development include Interferon Regulatory Factor 8 (IRF8) [7], a transcription factor key in cellular lineage commitment, and Spalt-like Transcription Factor 1 (SALL1) [8], a transcriptional regulator associated with maintaining microglial identity. Bone marrow derived monocytes, that enter the brain before the formation of the blood–brain barrier, are capable of differentiating into microglia-like cells, but these ‘microglia’ lack SALL1 expression, in addition to differing in morphology [9]. Thus, they can be distinguished from those derived from the internal pool within the CNS. Microglia are unevenly distributed during embryogenesis [10], and distinct populations arise during the early phases of development [11]. This begins with a proliferative population that is not found in adulthood [12], a Cd11c expressing group that acts as a source of Insulin-like growth factor 1 (IGF1) during myelination of the white matter tracts [13,14,15]. Additionally, so-called ‘Axonal Tract Microglia’ found during axonal tract development feature an amoeboid morphology and genes associated with lysosomal activation [12], along with multiple other phenotypes. With such diversity of microglia and distinct populations even in the early stages of development, it is unsurprising that there should be such heterogeneity throughout life. They are closely apposed to neurons and synapses, and also form contacts with astrocyte processes within the synaptic region [16,17]. It has been estimated that microglia make up 5–12% of the brain’s cell content [18]. In mice, it has been demonstrated that the transcriptomic phenotype of microglia is heavily dependent upon the local microenvironment [19], and more recently these findings have been replicated in humans [20]. This is significant given the regional heterogeneity of microglia, and how microglial activation in response to injury or insult can be localised rather than encompassing the entire brain. Of note, Böttcher and colleagues [20] noted differences in the expression of the G-protein coupled purinergic receptor P12Y, and IRF8, when comparing fresh human microglia with those obtained post mortem, suggesting differences in collection methods may be an important factor to consider when defining microglial diversity.

## 3. Microglial States and Functions

**Figure 1:**
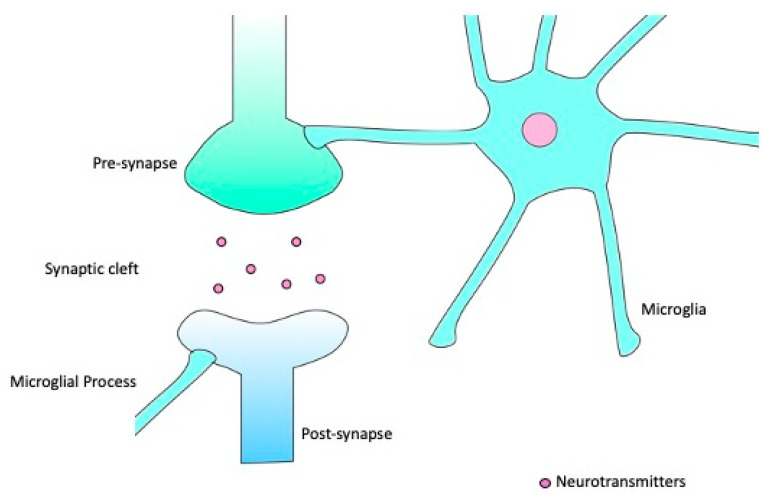
Microglia can extend and retract their processes, making brief contacts with synaptic structures whilst in the surveillance state.

The term ‘resting’ microglia is actually something of a misnomer, as even in this state microglia are highly dynamic [21]. In this ‘surveillance’ state, typified by a highly ramified morphology, microglia extend and retract many processes capable of forming contacts with other cells and structures [22], including synapses [23], enabling a continuous monitoring of the brain microenvironment. This continuous monitoring activity is enabled by their close physical relationship with neurons, particularly at the synapse. In this ‘resting’ state, whilst the soma is indeed ‘at rest’ and immobile, the processes are continually in motion, extending and retracting to form brief contacts with synapses, as depicted in Figure 1 [22,23]. The frequency of these contacts appears to be connected to neuronal activity [17,24], and such interactions occur more frequently with smaller dendritic spines. Smaller spines are also more frequently eliminated than their larger counterparts [16]. The contact between microglia and synapses is of great importance for synaptic pruning [25], another function carried out by resting or surveying microglia. Synapse formation in the early stages of life vastly exceeds what is necessary, and so in order for normal circuit development and function to occur, some of those excess synapses must be removed [26,27]. A selective partial phagocytosis (or trogocytosis) of presynaptic structures by microglia has been demonstrated [28]. It has been suggested that microglia interact with immature synapses through the classical complement cascade, with C1q (Complement component 1q) and C3 (Complement component 3) being expressed on synapses [29,30], although questions remain to be answered as to the precise mechanisms involved in pruning.

In what should be considered an over-simplification, it was suggested that upon encountering Damage Associated Molecular Patterns (DAMPs) or other foreign material, microglia enter a ‘classically’ activated or ‘M1’ state, leading to a drastic increase in the production of inflammatory cytokines including IL-6 (Interleukin 6), IL-1β (Interleukin 1β), TNF-α (Tumour Necrosis Factor α), and IFN-γ (Interferon γ), as well as Nitric Oxide (NO) and Reactive Oxygen Species (ROS) [31,32]. In vitro, these cells have also been shown to express increased levels of Fc γ receptors, alongside Major Histocompatibility Complex (MHC) class II and Cluster of Differentiation 86 (CD86), increasing their capacity for immune cell interaction and pathogen presentation [33,34,35,36]. However, whilst the concept of an ‘M1’ microglial phenotype like that seen in M1 macrophages may appear a reasonable one, with supportive in vitro evidence, in vivo evidence has been less forthcoming, possibly as a product of the heterogeneous and dynamic nature of these cells [37].

The M2 or ‘alternative activation’ phenotype is conventionally considered to be anti-inflammatory in nature, associated with wound healing and phagocytosis of cellular debris. However, it is a far less distinct classification than that of the M1 microglia [37]. Arginase 1, an enzyme with the capacity to diminish NO production [38], has been suggested as a specific M2 marker [39,40], as has Found in Inflammatory Zone 1 (FIZZ1), a secreted protein encouraging the deposition of an extracellular matrix [41,42]. The idea of an alternative activation phenotype initially stems from activation of macrophages induced by Interleukin 4 (IL-4) and Interleukin 13 (IL-13) [41]. In more recent times, it has been recognised that a single cohesive M2 phenotype fails to reflect the diversity of microglial populations, and consequently, there has been a shift towards M2a-c sub-classifications. In this classification, the traditional alternative activation phenotype is designated M2a, alongside M2b for cells playing roles in immunoregulation, and M2c cells, which express Cluster of Differentiation 206 (CD206) and Interleukin 4 Receptor Subunit Alpha (IL-4Ra) and have neuroprotective functions [36,37,43,44]. However, the argument has been made that even these subcategories still reflect a vast over-simplification of diverse microglial reactive states, failing to account for potential overlap [45] (Table 1), and that the M1/M2 paradigm should be done away with all together [46,47].

Another microglial phenotype that has been described as being associated with neurodegeneration is that of Disease Associated Microglia (DAM) [56,64]. This phenotype has been described as possessing features of both M1 and M2 microglia, further undermining the concept of binary divisions of activated microglia. DAMs have been reported in various mouse models [65,66,67], and also in human tissues [56,68]. DAMs, lying somewhere on the spectrum of activated microglia, are generally accepted to present a de-ramified, amoeboid morphology, with downregulation of various homeostatic genes. Various genes are upregulated, including Triggering Receptor Expressed on Myeloid Cells 2 (TREM2), a multi-functional protein involved in lipoprotein and apolipoprotein binding as well as being linked to microglial activation, APOE, involved in cholesterol packaging, Major Histocompatibility Complex II (MHCII), required for antigen presentation, and Cluster of Differentiation 44 (CD44), a glycoprotein involved in cell adhesion, migration, and cellular interactions [57,58,59]. Significantly, this transcriptomic signature is distinct from that of neuroinflammation as induced by Lipopolysaccharide (LPS), or that of generalised neuroinflammation. DAMs also show a specific pattern of localisation to those areas that are particularly vulnerable to neurodegeneration such as the hippocampus whilst being absent from the cerebellum [56]. Microglia closely surrounding amyloid plaques in both human and mouse models were found to have increased APOE expression [59], corresponding to this phenotype. It has been posited that DAMs go through a two-stage activation process, with the switch from stage 1 to stage 2 mediated by TREM2 signalling [67,68,69], with Phosphoinositide 3 Kinase (PI-3K) and Mammalian Target of Rapamycin (mTOR), which are involved in survival signalling, having been linked to TREM2-mediated processes.

Aged microglia have also been described as ‘primed’ [62,70,71], whereby the threshold for the microglia to be triggered to an activated state is substantially lower, and this has been suggested to be under the control of mTOR-dependent translation [72]. Interestingly, mTOR signalling has been reported to be impaired by TREM2 deficiency [69]. Aged microglia demonstrate reduced process complexity and diminished arborisation [73], comparable to the more amoeboid morphology associated with activation [71]. Stimulation with IL-1β and IL-12 has been reported to induce microglial priming in mice [63], with Interferon γ having been implicated particularly in terms of microglial priming for ROS production [74]. Interferon γ has been suggested to be involved in ‘classic’ priming, which is neurotoxic in nature, whereas Toll-like receptors (TLR) 2, 3, and 4 are implicated in ‘alternative’ priming, argued to be neuroprotective [60,75], with this paradigm mimicking that of the original M1/M2 classical/alternative microglial activation. It remains to be seen if this division of priming will stand up better than that of activation.

Whilst historically focus has been on activated microglial phenotypes as are classically associated with neuroinflammation, which is thought to be key to neurodegeneration, there is a body of evidence suggesting that the central mediator may be another phenotype altogether.

## 4. Microglia and Senescence

**Figure 2:**
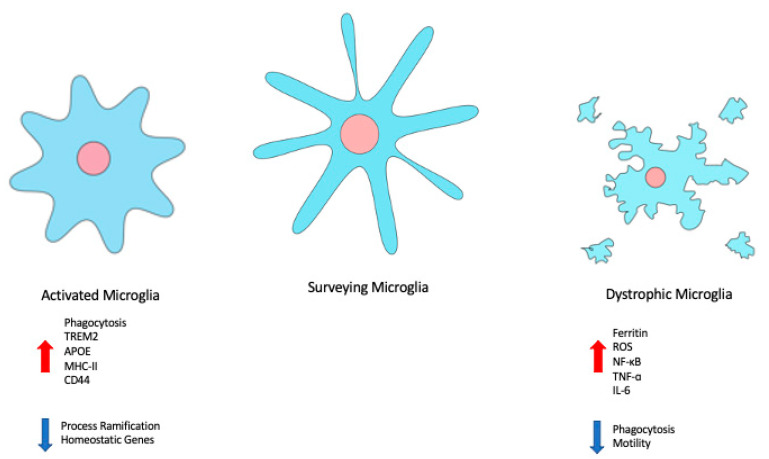
Changes in microglial state are associated with changes in morphology, gene expression, and behaviour. Disease-associated or -activated microglia tend to be more amoeboid in shape, with retracted processes, and demonstrate increased phagocytosis. Dystrophic or senescent microglia exhibit cytorrhexis, and a decrease in phagocytosis and motility.

The concept of cellular senescence originated with the discovery that cells in culture have a limited capacity for division [76], and has since then largely been studied in terms of its role in cancer [77] and ageing [78]. Microglia, unlike neurons, do have the capacity to divide and undergo replacement (although the time course over which this takes place remains controversial), but have also been found to undergo an ageing process. There are questions as to whether microglial senescence is a phenotype distinct from that of microglial ageing [79], or if this is a product of the differences between in vivo and in vitro studies. Senescence as a descriptor is sometimes used interchangeably with dystrophic. ‘Dystrophy’ now tends to refer more to morphological changes, whereas ‘senescence’ may be used to refer to specific secretory phenotypes, particularly associated with ageing [80]. Although specific markers for microglial senescence are yet to be established, there are certain morphological characteristics of dystrophy, such as deramification and retraction of processes, development of abnormal swellings in remaining processes, and cytoplasmic fragmentation or cytorrhexis [81], that could be considered identifying (Figure 2). These features have been observed in healthy but aged brains [82,83], although it has also been suggested by a study using human brain tissue that senescent microglia are exclusively a disease-associated phenotype [84]. Senescent cells are still metabolically active, and capable of inducing changes in their environment through secreted molecules, in what has been termed the Senescence-Associated Secretory Phenotype (SASP) [54,85,86], although it has also been referred to as the Senescence-Messaging Secretome (SMS) [77]. Crucially, it is suggested that this senescence phenotype can be transmitted between cells, and even between different cell types [54,87]. PInk4a is a marker of cell cycle arrest [88,89], and so could be considered a marker of cellular senescence when defined as arrested. β-Galactosidase expression, in particular so-called Senescence-Associated β-Galactosidase (SA-β-Galactosidase), has also been suggested as a senescence marker; however, it has also been observed in hippocampal neurons from 3 month old mice [90]. As a result, its validity as a unique senescence marker has been questioned. Senescence may also be associated with changes in energy metabolism, with these cells demonstrating a shift towards glycolysis [91,92,93,94], which is less efficient in terms of energy production than oxidative phosphorylation. This may provide an explanation as to why senescent microglia show a reduced capacity for carrying out processes such as phagocytosis.

SASP is a secretory phenotype, associated with increased secretion of pro-inflammatory molecules, and also those involved in processes of matrix-degradation [53]. Enhanced secretion of Tumour Necrosis Factor α (TNFα) and Interleukin-6 (IL-6) has been reported [95,96] in aged microglia. Mitochondrial dysfunction, and consequent defects in energy metabolism [97,98,99], along with enhanced Reactive Oxygen Species (ROS) production, are also noted features of SASP, and DNA damage [53]. Mitochondrial DNA damage in particular has been identified as elevated in aged microglia [100,101], along with telomere shortening [102]. The nature of SASP as a secretory phenotype complicates its characterisation, as there are perhaps more challenges associated with detecting and quantifying secreted proteins than their intracellular counterparts [103], but it seems likely that an SASP signature for microglia will eventually be described.

Senescent microglia have been detected in multiple brain regions [104], suggesting that this is not a phenotype specific to a single population of microglia, but rather a process that affects the vast majority of microglia. Whilst ageing may be a universal process, the associated signatures may differ in a regional fashion [105], a factor which SASP fails to account for. Replicative senescence, as a product of telomere shortening, has been reported in both rat and human microglia, including microglia isolated from patients with Alzheimer’s Disease (AD) [102,106]. Senescence as a product of DNA damage [107,108] and cellular stress has also been reported [109,110,111]. Senescence induced in the latter fashion has been referred to as Stress Induced Premature Senescence (SIPS) [110]. In addition to the reduced capacity for phagocytosis, senescent microglia display reduced motility, meaning that they are less able to migrate to sites of damage or debris [95]. Although senescent cells are highly heterogeneous, there are general classes of genes that are frequently associated with senescent phenotypes, including membrane trafficking, in agreement with the secretory aspect of senescence, and NF-κB [112,113]. Of note, it appears that senescence is a progressive phenotype, with changes occurring over time such that senescence could be divided into three stages, defined by changes in expression of interleukin isoforms, followed by interleukins and their receptors, and finally Matrix Metalloproteinases (MMPs) and their inhibitors [114].

Despite the fact that microglia do undergo turnover and replenishment, it seems that they are still vulnerable to the effects of ageing. Cell culture models have been used to demonstrate that ‘aged’ primary microglia (at 16 Days in vitro) are less capable than their younger counterparts of migrating towards and phagocytosing amyloid β oligomers and fibrils [115], showing a reduction in phagocytosis, thought to be linked to increased expression of CD33 and decreased expression of TREM2, and migration. This was also associated with increased MMP9, of note given that increased matrix metalloproteinase activity has been linked with senescent phenotypes [116,117]. Various transcriptomic phenotypes for aged microglia have been established [118,119,120], and found to include several of the genes associated with increased risk of neurodegenerative disease, including TREM2. Whilst the transcriptomic signature of aged microglia did not show an association with dementia diagnosis, a link with amyloid deposition was established, once again highlighting the potential importance of aged microglia in the development of neurodegenerative disease [119], along with several genes and pathways associated with SASP, but equally interesting, many other pathways, including DNA methylation and others not linked to SASP, suggesting that SASP alone may not be sufficient to describe the complexity of aged microglia. Interestingly, APOE ε2 was associated with a reduction of the aged microglia phenotype, perhaps suggesting a mechanism through which this haplotype may exert its neuroprotective effect.

Even though neuronal loss is a hallmark of ageing, it has been suggested that no such loss of microglia occurs [121]. The same study suggested the gene *Ctss*, which is expressed in all immune cells, was significantly increased only in aged microglia, once again suggesting that ageing is not a universally identical process. However, whilst microglia themselves do not appear to be lost in the ageing process, that is not to say that loss of function is not a concern for ageing cells, as a reduction in phagocytic ability is a particular concern in ageing microglia [83,95].

Downregulation of TGF-β signalling also indicates a loss of homeostatic function in aged microglia. However, there exists significant variation between published data sets. Such variation could be a product of differences in collection methods, or the aforementioned variation between species. The possibility should not be overlooked that a spectrum of aged microglia exists, even as is the case for microglial reactivity. The use of autofluorescence to distinguish between cell populations was significant in revealing the presence of lysosome-based storage defects in aged microglia [122], which is of interest given that excess iron storage and accumulation is such a key feature of aged or senescent microglia.

Dyshomeostasis of metal ions, particularly Fe, [123,124,125,126,127,128,129] has previously been established as a contributing factor to microglial dysfunction. Appropriate iron metabolism is of critical importance for energy production, as iron is a key co-factor for mitochondrial respiration [130], possibly implying a connection between iron dyshomeostasis and altered energy metabolism in senescent microglia [131]. Microglia are critical to iron homeostasis in the brain, taking up and storing iron molecules in the protein ferritin [132]. The expression of ferritin has been found to be increased in various neurodegenerative diseases [133], and has been explored as a potential CSF biomarker for predicting outcomes in Alzheimer’s Disease [134]. Increased brain iron could potentially contribute to increased oxidative stress [125,129], and microglia, in sequestering excess iron as a neuroprotective function, then themselves become subject to this damage. The argument has been made that free radical injury, as a product of oxidative stress, is a leading factor in development of microglial senescence [135]. Evidence of iron accumulation having a cytotoxic effect on microglia can be observed in the overlap between ferritin positive microglia and microglia displaying a dystrophic morphology in samples from human patients [126,129,136,137]. It has been suggested that so-called ‘M1’ macrophages and microglia may be more vulnerable to ferroptotic or iron-induced cell death due to their being enriched in inducible Nitric Oxide Synthase (iNOS) [138]. It is not the case that everything associated with iron metabolism is necessarily deleterious in impact; Transferrin (Tf) has been suggested to increase microglial phagocytosis in the presence of a demyelinating lesion [139], as well as reducing nitrite release in response to lipopolysaccharide (LPS) stimulation. The accumulation of iron may be linked to overexpression of Heme-Oxygenase 1 (HMOX1) in aged microglia, suggesting yet another potential marker requiring further investigation [140]. The expression of HMOX1 in microglia has previously been suggested as a potential mechanism for preventing inflammation in the brain [141], possibly as a result of cooperation with astrocytes and diminished expression of IFN-γ.

## 5. Microglia in Neurodegeneration

As is perhaps suggested by the existence of the DAM nomenclature for microglial activation [56,59], microglia are intimately involved in the pathology of neurodegenerative disease [58]. Reducing inflammation in neurodegenerative and adjacent diseases has been explored as a therapeutic strategy [142,143,144,145], and, when measured through the surrogate outcome of microglial activation, as an indicator of drug action [146,147]. Whether this is in terms of activated, inflammatory microglia or senescent microglia with corresponding loss of neuroprotective functions, microglia have been linked to every major neurodegenerative disease, with roles beyond pure inflammation. The concept of microglial involvement in neurodegenerative disease is not a new one, but our understanding of the role they play is constantly evolving.

Microglial senescence has been linked to the development of tau pathology in the early stages of Alzheimer’s disease [81,129,148], with dystrophic microglia displaying physical association with neurofibrillary tangles and neuritic plaques. It has been suggested that it may be a loss of microglial protection, as opposed to microglial activation, that could be a driving force in AD and other neurodegenerative conditions [126,127,135,149,150]. The dystrophic microglia found associated with amyloid plaques in the AD patient brain demonstrated positivity for ferritin [135,137], a calling card for microglial senescence, and yet another indicator of the potential toxicity to cells of iron overload. Dystrophic microglia have been visualised in AD brains by staining to reveal cytoplasmic fragmentation and disintegration [150], and there have been suggestions that patterns of microglial behaviour in the AD brain are extremely heterogeneous [151]. The argument has been made that senescent or dystrophic microglia are involved in the early development of AD, with activated or inflammatory microglia having a role to play much later in the disease course. Brain regions with fewer ferritin containing microglia demonstrate greater tissue iron accumulation [152], leading to increased ROS production through the Fenton reaction, suggesting that the build-up of iron in ferritin in microglia may initially function as a neuroprotective mechanism [153], with oxidative damage being absent from the microglia, but found in the damaged cells that they surrounded [152]. Interestingly, iron has been found to accumulate in microglia surrounding amyloid plaques in AD brains during clinical studies [154]. In addition, a loss of phagocytic ability, which is known to be a feature of senescent or aged cells, has been implicated in the diminished capacity of the brain for clearance of Amyloid β (Aβ) [75,95,115,155], paving the way for formation of amyloid plaques and their spread throughout the brain. It has been suggested that microglia become dystrophic or senescent as a result of exhaustion following their attempts to phagocytose and remove accumulating amyloid plaques [127]. Nije and colleagues [95] suggested that phagocytosis of Aβ by microglia may also be involved in distributing amyloid oligomers throughout the brain, again contributing to the spread. Microglia of an inflammatory phenotype have been described in APP/PS1 mice [91], with these cells showing a distinct tendency towards iron retention, but also other metabolic changes, including a shift away from oxidative phosphorylation towards glycolysis. TREM2 knockout or dysfunction has been linked to a shift towards glycolytic metabolism and away from the more efficient oxidative phosphorylation [69,156,157]. An important finding in terms of directing future research into the role of microglia in AD is that there are significant differences in microglial behaviour and gene expression in AD compared to that observed in mouse models [67]. Given the continuing debate as to whether microglia in AD are activated, dystrophic or a mixture [151], much work remains to be done.

Microglial response has also been implicated in Amyotrophic Lateral Sclerosis (ALS) [158,159,160], largely in terms of microglial activation and inflammatory responses. More recently, attention has turned to the role of senescent microglia in ALS, with microglia from the SOD^G93A^ mouse in culture expressing classical senescence markers, including p16^INK4a^, p53, and MMP1, suggestive of SASP [52]. This coincided with motor neuron loss in the model organism. Soluble iron is once again implicated in neurodegeneration, as its intracellular accumulation in microglia has been suggested to enhance activation of aconitase 1 (ACO1) and tumour necrosis factor α converting enzyme (TACE), leading to increased TNF-α stimulation of glutaminase-C (GLS-C), with resultant induction of glutamate release by microglia, contributing to excitotoxicity [161]. TNF-α, IL-1β, MMP12, and several other genes associated with a senescent phenotype have been found to be increased in ALS brains, again indicative of the potential for the involvement of microglial senescence in pathology [47].

The involvement of microglia has also been demonstrated in Multiple Sclerosis (MS). Senescent microglia demonstrate an impaired ability to clear myelin debris in mouse studies [162]. As it is necessary for myelin debris to be cleared before remyelination can occur [163], microglial senescence could be argued to force demyelinated lesions to remain so [164]. The introduction of ‘younger’ CNS immune cells (i.e., microglia) provides a boost to remyelination [165], implying that aged microglia are less capable of mounting an appropriate response and so create an environment of increased vulnerability to the diseased state. Lysosomal processing defects have also been implicated [166].

A hallmark of senescent or dystrophic microglia in the ageing brain is the accumulation of iron [55,167,168,169], with documented negative impacts on cognition. The accumulation of iron is noted in regions that are particularly vulnerable to pathology in Parkinson’s Disease (PD) [170], with iron loaded, dystrophic microglia being found associated with Lewy Bodies. The deposition of iron in microglia has been linked with severity of cognitive deficits in PD [171], and has also been demonstrated as a consequence of exposure to α-synuclein fibrils [172], the latter raising interesting questions as to where iron dyshomeostasis lies in the chronology of PD development. Iron exposure has been noted as a risk factor for PD in clinical studies [173,174]. Microglia are of critical importance for the clearance of α-synuclein via an autophagic mechanism dependent on TLR4 (which has been linked to ‘alternative’ microglial priming) [175], reacting specifically to the oligomeric form and not monomers [61,176]. Oligomers have also been shown to activate TLR2 [177]. There is significant evidence for the role of activated microglia in PD linked to α-synuclein [178]; however, senescent microglia should not be ignored. Dystrophic, dysfunctional microglia have been linked to neurodegeneration in brain samples from patients with Dementia with Lewy Bodies [126]. In addition, model senescent microglia have been shown to induce increased transcription of α-synuclein [179] via TNFα, and critically, aggregation and tetramer formation. The role of microglia as secretory cells also has no small part to play, with exosomes containing α-synuclein released by microglia suggested to enhance transmission, as well as aggregation of the protein in neurons [180,181].

## 6. Modelling Microglial Senescence

Several model systems have been developed for the study of the senescent microglial phenotype. In vivo, repeated injections of phorbol myristate acetate into the substantia nigra of rats [182] have been reported to induce a senescent phenotype of microglia, as indicated by increased activity of β-galactosidase and p21 induction. However, with any animal study, there will inevitably be ethical concerns, as well as issues presented by the extended time course required for such work. A senescent-like phenotype has also been reported in vitro as a result of treating cells with the corticosteroid dexamethasone [183], although in this model, expression of inflammatory genes and cytokine release were both decreased, which is not representative of the phenotype of aged microglia. Use of the chemotherapeutic drug doxorubicin [184] has produced a similar phenotype, raising questions about the long-term consequences of chemotherapy on the brain for cancer patients. However, this is perhaps not the most representative model of how microglial senescence might arise in the majority of individuals as part of the normal ageing process. The use of ionising radiation to induce senescence presents a similar quandary [185].

Supplementation with iron [179,186] has been demonstrated to induce cellular senescence, recapitulating the accumulation of iron in the ageing brain [80,124,187,188], in which not all cell types are equally affected [167,189,190]. Microglia show the greatest tendency for iron accumulation [190], which becomes particularly apparent when they are cultured with other cell types, or in organotypic slice cultures. Although ferritin is also found in neurons and oligodendrocytes, ferritin found in microglia is richer in Light-chain (L-chain) [188,191,192], which is optimised for the long term storage of ferric iron, than that found in other cell types. Significantly, a feature unique to dystrophic microglia in the ageing brain is the excessive accumulation of iron [124,136,137]. Of note, it has been demonstrated that these model senescent microglia are capable of inducing neurodegenerative-like changes in neuronal cells in culture models [179,186]. This model has been extensively validated, in both primary mouse microglia and human microglia, demonstrating significant retention of iron, morphological alterations, as well as increased release of ROS and cytokines (including IL-1β, TNF-α, and Interferon γ), suggestive of SASP or a similar phenotype. Increased ER stress and decreased autophagy, major indicators of cellular stress, were also validated by monitoring of mTOR and eukaryotic translation initiation factor 2A (eIF2a) and its phosphorylated form. A reduction in release of Insulin Degrading Enzyme (IDE) was also observed [186], resulting in increased levels of amyloid β, suggesting a potential mechanism by which senescent microglia may contribute to the development of advanced AD pathology.

Given the increasing evidence for the role of microglia in ageing, and in particular, neurodegenerative diseases, and the significance of iron in microglial ageing and dystrophy, it is not surprising that iron (and particularly, iron associated with microglia) should be linked to neurodegeneration. Microglia containing high levels of iron have demonstrated increased expression of the glycolytic enzyme PFKFB3, along with increased iron retention [91], suggesting that these cells take on a more glycolytic phenotype. Given the reduced energy efficiency of glycolysis as compared to oxidative phosphorylation, this could potentially explain the reduced phagocytic capacity of these microglia. An intriguing suggestion for future study is that that the accumulation of iron in microglia could function initially as a protective mechanism, to defend the brain from the neurotoxic effects of excess iron, with the eventual consequence that microglia themselves begin to experience those effects, leading to dystrophy and senescence [193].

## 7. Eliminating Microglia?

There is some precedent for the toxicity of senescent cells, with several studies identifying that the elimination of senescent cells as potential mechanisms for countering their deleterious effects (reviewed in [194]). Whole body elimination of senescent cells expressing p16^INK4a^ in mice modified to allow the genetic destruction of these cells by expression of the *INK-ATTAC* transgene [89] following administration of the synthetic drug AP20187, with a resultant improvement in cognitive function [195]. The use of a novel transgene to allow targeting of senescent cells has also been achieved on a progeroid or ageing-prone background, the BubR1 mouse [89], delaying the onset of age-related diseases, or even limiting their progression in older animals where diseases were already established. In a transgenic mouse model of tau-mediated neurodegeneration, the *INK-ATTAC* transgene was also used to eliminate p16^INK4a^ positive senescent astrocytes and microglia [196], which prevented gliosis and tau deposition and hyperphosphorylation, as well as cortical and hippocampal neuron degeneration.

An alternative approach has exploited metabolic changes in aged human myeloid cells [197], namely, macrophages and microglia, focussing on ‘resetting’ or reprogramming the cells rather than depleting them entirely. In aged myeloid cells, Prostaglandin E_2_ (PGE_2_), a lipid messenger, signals through its EP2 receptor, increasing glucose sequestration into glycogen, and thus reducing glucose flux throughout the cell. Inhibition of this signalling in aged mice reduced brain inflammation, as well as reversing defects in hippocampal synaptic plasticity and spatial memory [197,198]. This therefore suggests that these cellular defects in aged microglia may not be permanent, and by reversing these defects, the surrounding neurons may also be rejuvenated. In addition, these findings indicate the critical importance of energy metabolism within the brain.

Specific depletion of microglia by targeting of Colony Stimulating Factor 1 Receptor (CSF1R) has been utilised in mouse models, for the purpose of impeding the propagation of phospho-tau, such as is observed in AD [199]. However, even as this demonstrates the principle of specifically targeting microglia, such a large scale depletion of the cell type is likely to be of limited practical benefit in a clinical setting. CSF1R signalling has been shown to be essential for microglial survival and viability [200], and its inhibition in mouse models has been shown to eliminate 99% of CNS microglia. Inhibition of CSF1R, then removal of this inhibition for 1 week, was demonstrated to allow ‘repopulation’ of microglia [200,201,202]. Whilst CSF1R knockout mice are completely lacking in microglia, and die prior to attaining adulthood [203,204], the elimination and repopulation of microglia via this method in adult mice appears to trigger no cognitive, motor function, or behavioural deficits [200,201]. The repopulating microglia are reported to be similar to control microglia in mRNA gene expression and responsiveness to LPS, with initial morphological differences, namely, a more amoeboid shape with fewer, shorter processes, resolving rapidly. However, it remains to be seen if this approach would be so successful in the larger, more complex human brain, where cell volume is substantially greater than in the mouse. Microglial elimination and repopulation in an aged mouse model was shown to improve cognition, particularly spatial memory, concurrently increasing density of synaptic spines and neurogenesis [202]. These processes are diminished in the aged brain, demonstrating benefit not only to the microglia but also to the surrounding neurons.

Targeting and eliminating or reprogramming aged or senescent microglia clearly holds potential for reversing the impact of ageing on the brain, and much has already been learned from such techniques [194]. However, at the present time, it remains unclear how these techniques might be translated into benefit in human patients. An ideal outcome would be the ability to target specifically aged, senescent, and neurotoxic microglia and eliminate them from the brain without the requirement for genetic manipulation and transgene expression. Efficacy of such a technique may well be improved by more specific identification and targeting of senescent microglia, which would require the identification of a unique, specific marker.

## 8. Concluding Remarks

It is important to remember that SASP represents only one senescence phenotype, where the term ‘senescence’ encompasses a broad range as heterogeneous as microglia themselves. This heterogeneity may also contribute to the differences in microglial behaviour in vitro versus in vivo, which have thus far posed a significant obstacle to translational research, and yet have only recently been recognized.

It is clear that depletion, replacement, or rejuvenation of aged microglia could be a significant avenue for further exploration in the arena of cognitive decline and age-related neurodegeneration. However, existing techniques have relied upon transgene expression, or do not exclusively target microglia, meaning it is at present unclear how they might be translated to the clinic.

It is by now well known that microglia are a massively diverse, heterogeneous population, which will only complicate the identification of suitable markers for senescence. However, it has recently become apparent just how significant methods of obtaining and preparing microglia for investigation may be [20], presenting yet another confounding variable to the interpretation of the variation in published data. This heterogeneity is also likely to be significant for neurodegeneration, given the regional specificity of microglial senescence or activation and associated pathologies in several diseases [56]. The value of the phagocytic capacity of microglia in clearance of debris and protein aggregates is significant in at least three major neurodegenerative diseases (AD, PD, MS), and the loss of this ability during cellular senescence, whether this is SASP or otherwise, is a potential mediator of neuropathology.

In order to make further progress in characterising and understanding senescent microglia, it is of critical importance that these cells can be clearly identified, which requires a reliable marker. In order for such a marker to be identified, it is even more important that a representative model be established, such as by loading cells with iron [179,186]. The effects of this model should be replicated in human cells, not only in mouse or zebrafish, given the heterogeneity of microglia between species. Additionally, further work is required to investigate to what extent in vitro senescence phenotypes such as SASP are replicated in vitro, and fully characterise the phenomenon of microglial senescence. By identifying a consistent senescence marker, ideally one that is unique to microglia, a more targeted elimination of potentially neurotoxic cells may be possible, building on techniques already established to generate a mechanism of true translational value.

## Figures and Tables

**Table 1:** A variety of markers have been proposed for the various non-resting microglial states. The existence of overlap between markers for different states contradicts the idea of the binary ‘M1/M2’ paradigm. CCL2 (Chemokine Ligand 2); STAT3 (Signal Transducer and Activator of Transcription 3); CSTD (Cathepsin D); LPL (Lipoprotein Lipase); CLEC7a (C-type lectin domain containing 7a); TYROBP (TYRO protein tyrosine-kinase bindin protein).

	‘M1’	‘M2’	Senescent/Dystrophic	DAM	Primed
Cytokines	IL-1β, TNF-ɑ, IFN-γ, IL-6	TGF-β, IL-4, IL-13, IL-4Ra, IL-10	TNF-ɑ, IL-6		IL-1β. CD14
Chemokines	CD11b	CD206	CCL2	CD9, CD11c, CD14, CD44, CD86	
Membrane Receptors and Channels	MHC-II, Fc-γ, Kv1.3		MHC-II, Kv1.3	TREM2, AXL, CLEC7a	MHC-II, TLR-1, TLR-2, TLR-4, TLR-7
Transcription Factors and Growth Factors	NF-κB, IRF1, STAT3		NF-κB, VEGF		
Enzymes		ARG1	MMP1, SA-β-GAL	CSTD, LPL	
Other	ROS, NOS	FIZZ1, Ym1/2	Ferritin, p16^INK4a^	TYROBP, APOE, CST7	ROS
References	[42,48,49]	[37,39,41,42,50,51]	[52,53,54,55]	[56,57,58,59]	[60,61,62,63]
